# Larotrectinib as an Effective Therapy in Congenital Infantile Fibrosarcoma: Report of Two Cases

**DOI:** 10.1055/s-0042-1748866

**Published:** 2022-06-25

**Authors:** Lucas Moratilla Lapeña, Maria Carmen Sarmiento Caldas, Carla Ramírez, María San Basilio, Paloma Triana Junco, Lara Rodríguez-Laguna, Victor Martínez-González, Elena Marín-Manzano, Antonio Perez-Martinez, Juan Carlos Lopez-Gutierrez

**Affiliations:** 1 Department of Pediatric Surgery, Hospital Universitario La Paz, Paseo de la Castellana, Madrid, Spain; 2 Institute of Medical and Molecular Genetics (INGEMM), Hospital Universitario La Paz, Madrid, Spain; 3 Department of Vascular Surgery, Hospital Universitario La Paz, Paseo de la Castellana, Madrid, Spain; 4 Department of Pediatric Haemato-oncology, Hospital Universitario La Paz, Madrid, Spain; 5 Department of Pediatric Surgery, Division of Vascular Anomalies, La Paz Children's Hospital, Madrid, Spain

**Keywords:** congenital infantile fibrosarcoma, larotrectinib, children tumor

## Abstract

Congenital infantile fibrosarcoma (CIF) is a rare tumor in children that occurs in the first years of life. It usually arises in the extremities but some cases affect the trunk, neck, abdomen, or retroperitoneum. Surgical resection has been traditionally the treatment of choice but the development of genomic analysis and targeted therapies has shed light on new therapeutic options.

We present two patients with a congenital mass, one in the abdominal cavity (1-month-old) and the second in the left lower extremity respectively (2-months-old). In both cases, the clinical and radiological findings showed heterogeneous masses with rapidly progressive growth. MRI in the first patient exhibited an abdominal mass surrounding the aorta and inferior vena cava associated with a giant infrarenal aortic aneurysm. CT-guided biopsy was performed with pathological findings of fibrosarcoma and *ETV6-NTRK3* gene fusion. The second patient underwent open biopsy also with histopathological diagnosis of fibrosarcoma and the same mutation in the *TRK* gene ( *NTRK3* ). Targeted therapy with a specific TRK inhibitor, larotrectinib, was started in both patients. Periodical controls were made by ultrasound or MRI, and after a few weeks of treatment, both children showed significant decrease in the mass. By the second and third months after starting the treatment, both tumors disappeared. The first patient is now 15-months-old and the second one is 8-months-old.

Larotrectinib is a novel targeted therapy with excellent results in CIF but long-term outcomes are limited to establish it as a gold standard treatment.

## Introduction

Congenital infantile fibrosarcoma (CIF) is a rare tumor that arises from soft tissue. Its incidence is 5 cases per 1,000,000 infants and mainly arises from the extremities and the trunk. 1 2 3 The *NTRK* mutation seems to be the cause of tumor development, the most predominant being the *ETV6-NTRK3* gene variant. It has a good prognosis having 5-year overall survival rates of 90%. Surgery remains the gold standard treatment but when it is not feasible, neo-adjuvant chemotherapy maybe effective. 1 3 4 Recently targeted therapies had been used to treat locally advanced and chemotherapy-resistant sarcomas with good results. 5 6 7 We report two cases of non-surgical treatment in two neonates.

## Case Report

### Case 1

**Fig. 1:**
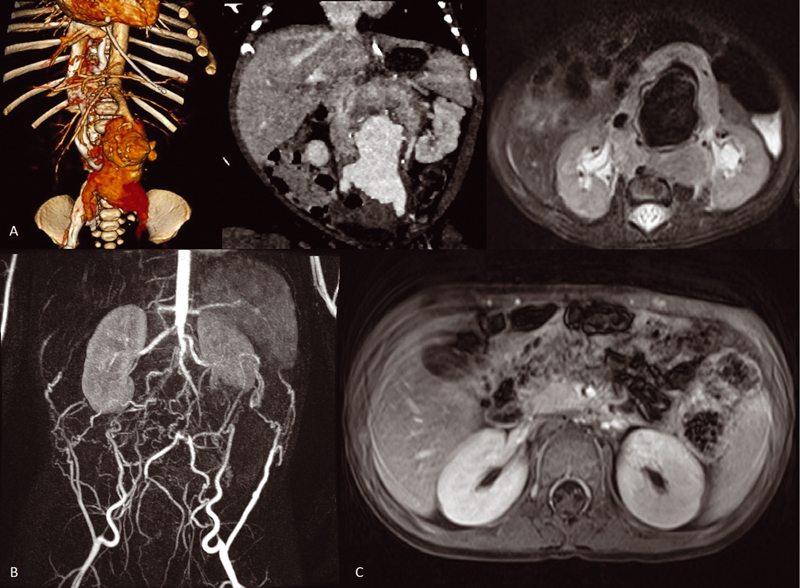
**(A)** Diagnostic images of a retroperitoneal mass that encompass the major abdominal vessel and an infrarenal aorta aneurysm. **(B)** Angio MRI that demonstrates collateral circulation after infrarenal aortic ligation. **(C)** Absence of tumor mass after treatment with larotrectinib.

A female newborn at 40-week gestational age with prenatal diagnosis at 35 + 4 gestational age of abdominal vascular anomaly was referred for evaluation. Physical examination revealed an abdominal mass. An MRI and angio-CT were performed with suggestive findings of a retroperitoneal mass encompassing major abdominal vessels and aneurysmatic dilatation of the abdominal aorta ( Fig. 1 ) with measures of 6.4 × 4.8 × 9.4 cm. After a CT-guided biopsy, pathological results were consistent with *ETV6-NTRK3* mutated CIF and treatment with specific TRK inhibitor (larotrectinib) was initiated at age of 2 months at a dose of 100 mg/m ^2^ twice daily for 1 year. Excellent response to the treatment was observed with total regression of the retroperitoneal mass by the third month after onset treatment ( Fig. 1 ). Despite total regression of the mass, the abdominal aneurism grew, so surgical excision with infra renal aortic ligation was made after an occlusion test with good tolerance. The patient is now 15 months old and is in her seventh month without treatment with no radiological or clinical evidence of tumor recurrence doing normal life.

### Case 2

**Fig. 2:**
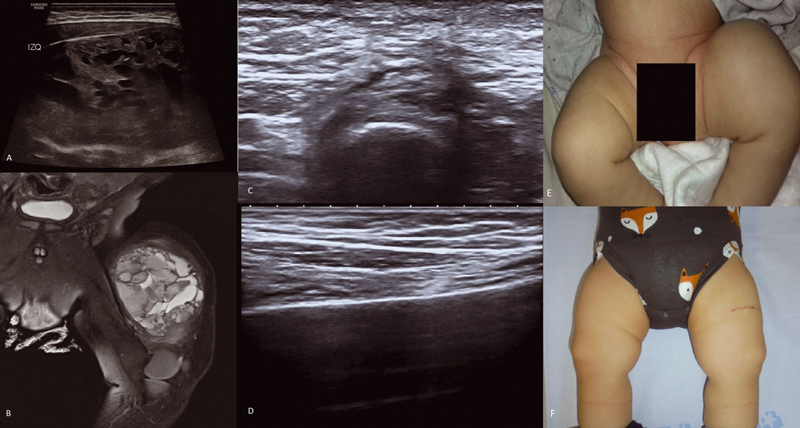
**(A)** Ultrasound of the left lower limb with findings suggestive of possible hematoma of the muscle. **(B)** MRI shows soft tissue tumor of the left lower limb. **(C)** and **(D)** demonstrate the absence of evidence of residual tumor on ultrasound. **(E)** Left lower limb pre-treatment. **(F)** Left lower limb after treatment, biopsy scar is seen.

A 2-month-old male child was referred to our institution with a lower extremity mass, which had started to grow in the first month of life. An MRI was made after an ultrasound with suggestive findings of possible hematoma. MRI revealed a soft tissue mass (5.1 × 5.6 × 6.9 cm) compatible with possible vascular malformation without being able to rule out other possibilities such as rhabdomyosarcoma ( Fig. 2 ), so an open biopsy was made. During the biopsy, intensive bleeding was observed. Anatomopathological findings were conclusive with CIF and *ETV6-NTRK3* translocation. Larotrectinib was initiated at 3 months of age at a dosage of 100 mg/m ^2^ twice daily. Periodical US controls were made showing the total remission of the tumor by the second month of treatment ( Fig. 2 ). The patient is actually on his eighth month of treatment without any evidence of radiological tumor recurrence.

## Discussion

Despite being a rare tumor, CIF treatment has great survival rates and despite its high morbidity associated with surgical excision, which remains the gold standard treatment. When radical resection is unfeasible, neo-adjuvant chemotherapy using vincristine, actinomycin, and cyclophosphamide shows responses, allowing the posterior excision of the tumor. 1 4 Other reported cases where resistant to the treatment or had serious complications as neutropenia. 7 8

Recently, genomic studies have been conducted allowing to know the molecular origin for these tumors. Translocation t(12;15)(p13;q25) with the transcript ETV6-NTRK3 is a recurrent mutation in this tumor; so, genetic studies should be done in all these patients. Targeted therapy for this mutation had great results in adult's sarcomas; however, there was no evidence of effectiveness in children until the first open-label, multicentric study was done. 6 This phase 1 study demonstrated that larotrectinib was safe and induced tumor sustained regression in over 90% of children with the *TRK* gene mutations and could recommend a dose of 100 mg/m ^2^ .

Since the publication of this study, a few more cases have been published. The first ones only had partial remission of the tumor allowing posterior surgical treatment 5 but posterior works published complete response to the treatment. 2 7

Those findings, supported by our two cases, shed light on the treatment of CIF, avoiding surgical treatment with a rapid decrease in the tumor size. More studies are needed but the effectiveness and safety of larotrectinib can make it as the targeted therapy in the mainstay treatment of *TRK* mutated tumors.

## Conclusion

Larotrectinib is a novel targeted therapy with excellent results in CIF but long-term outcomes are limited to establish it as a gold standard treatment.

## Notes

New Insights and the Importance for the Pediatric Surgeon The mainstay treatment of congenital infantile fibrosarcoma is surgical excision but the new target therapies could make this change by avoiding morbidity and mortality associated with surgical treatment.
